# Assessing the Presence of IgG Antibodies against Influenza Viruses in Neonates after Maternal Vaccination and Factors That May Affect the Transplacental Transfer

**DOI:** 10.3390/diseases11040166

**Published:** 2023-11-10

**Authors:** Chrysoula Taskou, Antigoni Sarantaki, Vasiliki Ε. Georgakopoulou, Gerasimos A. Spyratos, Panagiotis V. Drossos, Georgios Daskalakis, Apostolos Beloukas, Aikaterini Lykeridou

**Affiliations:** 1 Midwifery Department, University of West Attica, 11521 Athens, Greece; esarantaki@uniwa.gr (A.S.); klyker@uniwa.gr (A.L.); 2 Department of Infectious Diseases-COVID-19 Unit, Laiko General Hospital, 11527 Athens, Greece; vaso_georgakopoulou@hotmail.com; 3 Biomedical Sciences Department, University of West Attica, 11521 Athens, Greece; makisspyratos@hotmail.com (G.A.S.); pdrossos@uniwa.gr (P.V.D.); 4 Labogen S.A. Laboratories, 11143 Athens, Greece; 5 1st Department of Obstetrics and Gynecology, Alexandra Hospital, National and Kapodistrian University of Athens, 11528 Athens, Greece; gdaskalakis@med.uoa.gr; 6 National AIDS Reference Centre of Southern Greece, Department of Public Health Policy, University of West Attica, 11521 Athens, Greece

**Keywords:** maternal vaccination, influenza vaccines, influenza antibodies, IgG transplacental transfer

## Abstract

Special populations, particularly pregnant women, are uniquely susceptible to infectious diseases due to alterations in their immunological, respiratory, and cardiovascular systems during gestation. Influenza infections during the perinatal period have been associated with more severe maternal and perinatal outcomes, underscoring the critical importance of vaccination data for pregnant women. According to the World Health Organization (WHO), all pregnant women and those of childbearing age should receive the inactivated influenza vaccine, irrespective of their pregnancy stage. This study aimed to elucidate factors influencing neonatal antibody presence following maternal influenza vaccination. Conducted through convenience sampling in Athens, Greece, this study involved 78 pregnant women who received flu vaccinations. The participants completed questionnaires covering demographics, obstetric history, attitudes toward influenza vaccination, and knowledge about the influenza virus and pregnancy vaccination. Blood samples were collected from 83 neonates to assess IgG antibody presence. Five of the surveyed women had twin pregnancies. The statistical analysis employed IBM SPSS-Statistics version 26.0. This study revealed the presence of positive influenza A and B antibodies in neonates following maternal immunization. Furthermore, it identified factors such as the gestational week and timing of vaccination during pregnancy that influenced the transfer of antibodies from mother to fetus. These findings offer valuable insights for healthcare professionals to provide informed recommendations on influenza vaccination during pregnancy and empower expectant mothers to make informed decisions about the benefits of immunization.

## 1. Introduction

Special populations, such as pregnant women and children, are susceptible to severe viral infections due to pregnancy-induced changes in the immune system, which is adapted to tolerate the presence of a semi-allogenic fetus [1]. Consequently, viral infections during pregnancy pose a significant risk, leading to severe maternal illness, elevated maternal mortality, and various pregnancy complications, including premature labor, spontaneous abortion, and fetal congenital abnormalities, particularly affecting the cardiovascular and central nervous systems [2].

Immunization has emerged as a pivotal strategy for safeguarding both maternal and neonatal health during pregnancy [3]. International health authorities, including the World Health Organization (WHO), the Advisory Committee on Immunization Practices (ACIP), and the American College of Obstetricians and Gynecologists (ACOG), strongly recommend that all pregnant women and women of childbearing age receive the inactivated influenza vaccine, regardless of their pregnancy stage [4,5]. The Centers for Disease Control and Prevention (CDC) further advise that all pregnant or potentially pregnant women receive a licensed, age-appropriate inactivated influenza vaccine or the recombinant quadrivalent influenza vaccine during the influenza season [6].

In Greece, as in many European countries, comprehensive antenatal care programs recommend immunization for women planning to become pregnant or already expecting [7]. Several retrospective studies have demonstrated the safety of influenza vaccines during pregnancy. Recent systematic reviews endorsed by the WHO have unequivocally refuted any association between influenza vaccination and adverse effects on pregnant women, including risks of miscarriage, fetal death, maternal mortality, preterm birth, or fetal congenital abnormalities [8,9,10]. Maternal influenza vaccination is particularly crucial because it passes maternal antibodies to the developing fetus through the placenta, offering protection to the newborn against influenza, a disease for which there are currently no approved vaccines for infants under six months of age [11].

The World Health Organization (WHO) annually provides recommendations on the influenza strains to be included in vaccines for the forthcoming northern hemisphere flu season. Vaccine compositions are updated accordingly based on WHO and EU guidance. Seasonal flu vaccines typically contain influenza A-H1N1, influenza AH3N2, and influenza B virus (IIV3). The quadrivalent IIV influenza vaccine now includes an additional B virus strain to enhance protection. Quadrivalent inactivated influenza vaccines were first licensed in 2012.

Following influenza vaccination, the body initiates antibody production approximately two weeks later, thereby offering protection against the influenza strains used in vaccine production. Seasonal flu vaccines are designed to protect against the influenza strains projected to be most prevalent in the upcoming flu season.

Immunoglobulin G (IgG) constitutes the primary antibody class, accounting for approximately 75% of human serum antibodies and featuring the neonatal Fc receptor (FcRn) expressed on syncytiotrophoblast cells within endosomes. This receptor is responsible for the transplacental transfer of passive humoral immunity from mother to fetus [12]. FcRn’s role in transporting IgG across the placenta involves binding maternal IgG in moderately acidic endosomes within the villous tree, where normal pH is restored, facilitating its transport to the basal plasma membrane and subsequent release from the FcRn [13]. FcRn expression commences after the 13th week of pregnancy. Between weeks 17 and 22, only 10% of maternal antibody concentration is present in fetal blood, with a notable increase in transfer efficiency observed thereafter. The majority of transfers transpire during the third trimester, especially between 28 and 32 weeks, although various factors may influence this process. Intriguingly, cord blood levels of IgG increase significantly after the 36th week of pregnancy, yet many aspects of IgG transport across the placenta remain poorly understood.

Several factors have been identified that influence the transfer of placental antibodies. These factors encompass the mother’s overall and specific antibody levels, IgG subclass, maternal health conditions during pregnancy (e.g., HIV infection), placental pathologies, maternal hypergammaglobulinemia, and the nature of the antigen itself, particularly its immunogenicity for thymus-dependent antigens [14]. Notably, maternal age, weight, parity, and delivery method do not impact placental antibody transmission. Furthermore, the immunization of full-term and preterm newborns poses challenges due to their underdeveloped immune systems and limited immunological responses to vaccine antigens [15,16].

This study aims to determine the optimal timing for influenza vaccination during pregnancy by investigating factors that may affect transplacental antibody transfer, as evidenced by the presence of antibodies in neonates.

## 2. Materials and Methods

The Clinical Research and Ethics Committees’ boards of the Elena Venizelou and the Alexandra Public Hospitals approved this study (T59-Μ10/16-09-2020) as a minimal risk that could not practicably be performed without the waiver of consent. Prior to the commencement of this study, written informed consent was obtained from all participating mothers of neonates.

### 2.1. Settings and Study Population Inclusion and Exclusion Criteria

Population: This was an institutional review board-approved cohort study of women who delivered their newborns at the Elena Venizelou and Alexandra Public Hospitals in Athens, Greece, and had received the quadrivalent influenza vaccine (IIV4) in the time period up to 6 months before pregnancy until birth. Vaccinated women, ≥18 years of age, fluent in the Greek language, who gave birth at the above hospitals between 1 December 2020 and 31 August 2022, were eligible for this study. A total of 78 women who received the flu vaccination during pregnancy, reported no significant complications or illnesses during pregnancy, and delivered within the study period were finally enrolled.

Gravidas were excluded if they were vaccinated before conception or during the postpartum period, those who did not give informed consent to take part in the research, and did not give their permission for the neonates’ blood sera collection. Only the participants who fully answered the questionnaires were included in our study. Vaccine administration was ascertained via a search of the electronic medical record (EMR) system. Neonatal blood sera were available for antibody detection in 83 newborns.

### 2.2. Sample Collection and Preparation

Prior to full study participant and biological sample deidentification, demographic data (i.e., age, level of education, family status, etc.) and obstetric clinical history data (such as previous pregnancies, miscarriage history, etc.) were collected from self-administered questionnaires.

Two sections constituted the questionnaire. Section 1 included items on the demographic characteristics and obstetric clinical history of women participating in the research. The knowledge and attitudes of women toward the influenza virus and the influenza vaccination were covered in Section 2 (some representative questions were “what is influenza?”, “what is the recommended flu vaccine for pregnant women in Greece?”, etc.).

Preterm delivery was defined as less than 37 gestational weeks, and term delivery was defined as 37 or more gestational weeks.

### 2.3. Sampling Procedure and Antibody Detection

Upon the neonates’ first day of life or within 10 days post-birth, blood samples were meticulously collected from 83 neonates (5 out of the 78 women had twin pregnancies). Both twins from each of the five pairs were included in all analyses. Blood samples were collected in Clot Activator tubes. All blood samples were processed within 4 h, and serum was stored at 80 °C until further investigation.

The sera were subjected to qualitative analysis for the presence of IgG class antibodies against influenza virus A and influenza virus B using two commercially available enzyme-linked immunosorbent assays (ELISAs), namely the Demeditec Influenza Virus A IgG ELISA (DEINFG0290) and the Demeditec Influenza Virus B IgG ELISA (DEINFG0300), following the relevant instructions for use (IFUs) provided by the manufacturer.

Briefly, coated enzyme-linked immunosorbent assay plates (Demeditec Diagnostics GmbH, Kiel, Germany) were meticulously incubated with appropriately diluted sera samples for 1 h ± 5 min at 37 ± 1 °C, then washed three times with 300 μL of washing buffer. Subsequently, 100 μL of conjugate was dispensed into all wells, with the exception of the substrate blank well, and the plates were once again incubated for 30 min at room temperature. After an additional three washing steps, Sure Blue 3,3′,5,5′-tetramethylbenzidine substrate (TMB) was added to each well, and the plates were incubated in the dark at room temperature (20–25 °C) for exactly 15 min, leading to a color change due to enzymatic reactions. A total of 100 μL of 0.2 mol/L sulfuric acid (stop solution) was added to each well to stop the reaction. Finally, the absorbance at 450/620 nm was measured within 30 min. All sera were tested in duplicate, and the mean absorbance values were calculated. The results were classified as negative (<9 U), equivocal (9–11 U), and positive (>11 U) in accordance with the manufacturer’s instructions.

### 2.4. Statistical Analysis

The Kolmogorov–Smirnov test was used to determine whether the distribution of the variables was normal. For continuous variables with two groups that were normally distributed, the *t*-test was used. We also used the Kruskal–Wallis test for non-normally distributed variables with three groups. In addition, continuous variables are displayed as mean (standard deviation). Categorical variables have been analyzed and presented as absolute numbers (frequency percent) using Fisher’s exact or chi-square tests; *p*-values under 0.05 were regarded as significant. IBM SPSS Statistics version 26.0 (IBM, Armonk, NY, USA) was used for the statistical analysis.

## 3. Results

### 3.1. Demographics, Characteristics, and Obstetric History of Enrolled Pregnant Women

During the study period, a total of 78 women gave birth, with five experiencing twin deliveries. The largest proportion of participants (31 individuals, 39.7%) fell within the 35–40 years age group, and the overwhelming majority were of Greek ethnicity (71 participants, 91%). In terms of education, a substantial portion (46 individuals, 59%) held bachelor’s degrees, while with regard to family status, most participants (67 individuals, 85.9%) were married. Additionally, a significant number of participants (37 individuals, 47.4%) were employed in the private sector.

Concerning obstetric history, a notable proportion of pregnant women (34 participants, 43%) had no prior pregnancies. Ten individuals (12.8%) reported a history of miscarriage, while 14 individuals (17.9%) experienced premature birth. Moreover, 20 women (25.6%) had a history of chronic diseases encompassing conditions such as type 1 diabetes, gestational diabetes, thrombophilia, hypothyroidism, and systemic lupus erythematosus. Furthermore, 31 participants (39.7%) reported close contact with individuals at high risk for flu complications within their households, and 15 (19.2%) reported similar contact with high-risk individuals at their workplaces.

Regarding the timing of flu vaccination during the current pregnancy, the majority of participants (31 individuals, 39.7%) received the flu vaccine during the first trimester, with all vaccinations occurring during the flu season.

Table 1 presents a comprehensive overview of the demographic characteristics and obstetric history of the study participants.

### 3.2. Knowledge and Attitudes of Enrolled Pregnant Women Regarding the Influenza Virus and Influenza Vaccine

A noteworthy majority of participants exhibited a commendable level of awareness regarding the communicable nature of influenza (77 participants, 98.7%). Furthermore, a substantial portion acknowledged the potential severity of flu complications, recognizing the need for hospitalization (62 participants, 79.5%). Importantly, a considerable number of participants were cognizant of the heightened vulnerability of pregnant women to flu-related complications when compared to their non-pregnant counterparts (53 participants, 67.9%). Almost all participants (77, 98.7%) demonstrated an awareness of the recommendation and accessibility of free flu vaccinations for pregnant women in Greece, concurrently harboring the belief in the safety of administering the flu vaccine during pregnancy.

Concerning their past vaccination behavior, 27 participants (34.6%) reported having received the seasonal flu vaccine within the window of 6 months to 5 years preceding the current pregnancy. Furthermore, a substantial proportion (70, 89.7%) indicated that they had been adequately informed about the flu vaccine and the procedural aspects of obtaining it from healthcare professionals during the present pregnancy.

For a comprehensive overview of the knowledge and attitudes of pregnant women concerning the influenza virus and influenza vaccine, kindly refer to Table 2.

### 3.3. Association between Maternal Characteristics and Their Attitudes towards Influenza Vaccination

The present study aimed to investigate the association between pregnant women’s characteristics and their attitudes towards vaccination against influenza before pregnancy. A statistically significant disparity was evident in the proportion of participants who, within the timeframe of 6 months to 5 years before the current pregnancy, reported contact with high-risk individuals in their workplace and subsequently received a flu vaccine, in contrast to those who neither reported such contact nor received vaccination (9 out of 15 (60%) and 18 out of 63 (28.6%), respectively; *p* = 0.033). A comprehensive summary of these associations is provided in Table 3.

### 3.4. Pregnancies and Neonates’ Characteristics

Out of the 78 pregnancies observed, a minority (five, 6.4%) constituted twin pregnancies. Predominantly, deliveries were administered through Caesarean section (53, 67.9%). Among the neonates, the female sex was more prevalent (43, 51.8%). Additionally, a significant proportion of neonates (66, 79.5%) were delivered within the gestational age range of 37 to 42 weeks, and the majority (73, 88%) exhibited an appropriate birth weight.

**Figure 1:**
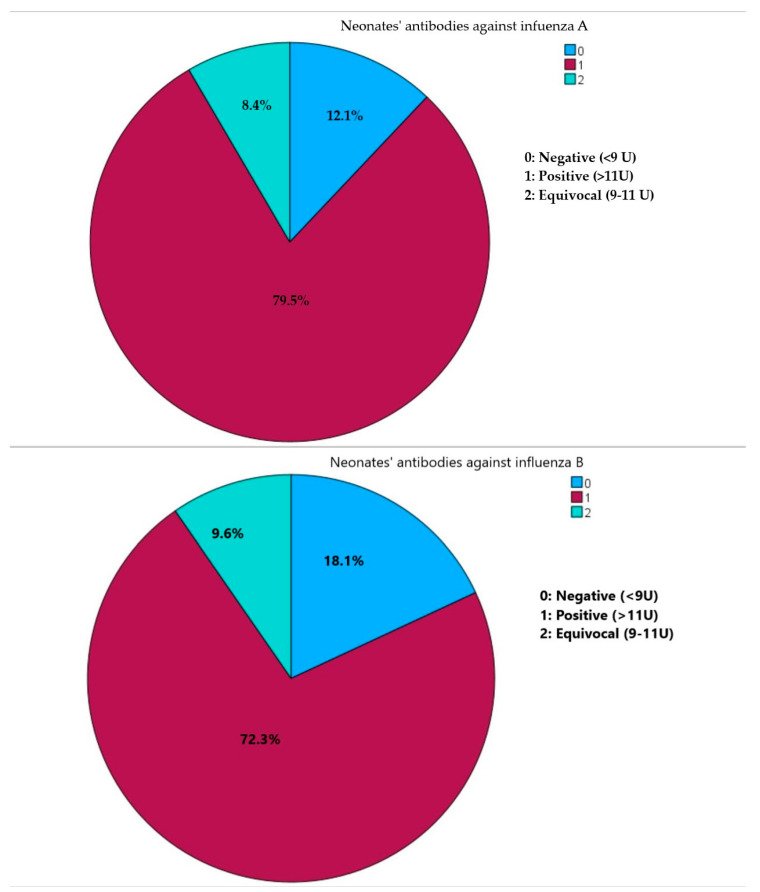
Presence of neonates’ IgG class antibodies against influenza virus A and influenza virus B.

Sample collection primarily occurred during the flu season, accounting for 46 (55.4%) samplings. In terms of qualitative antibody assessment, a substantial number of neonates exhibited positive antibodies against both influenza A (66, 79.5%) and influenza B (60, 72.3%). Furthermore, the majority of neonates (50, 60.2%) demonstrated positivity for both influenza A and B, while merely three neonates (0.04%) tested negative for both influenza A and B (Figure 1).

Table 4 provides a comprehensive overview of the characteristics pertaining to the pregnancies and neonates.

### 3.5. Association of Maternal and Neonatal Characteristics with the Presence of Neonates’ IgG Class Antibodies against Influenza Virus A and B

This study delved into the potential links between maternal and neonatal characteristics and the presence of antibodies against influenza A and influenza B. Our findings revealed a notably higher prevalence of positive results for influenza A antibodies within the neonates born between the 37th and 42nd week of gestation (*p* = 0.021), as well as among women vaccinated during the second trimester of pregnancy (*p* = 0.041). Furthermore, we observed a statistically significant association between the duration between vaccination and blood sampling and the presence of antibodies against influenza B. Participants who tested positive for influenza B antibodies exhibited a significantly shorter period between vaccination and blood sampling. These associations are displayed in Table 5 and Table 6.

### 3.6. Concordant IgG Detection in Twin Neonates

In our study, a total of five twin pregnancies were included, constituting a relatively small sample size. However, this limited cohort exhibited a noteworthy trend, where the twin neonates consistently displayed concordant detection rates for both influenza viruses. This observation implies a remarkable similarity in their immunological profiles. The results are displayed in Table 7.

## 4. Discussion

Influenza poses a significant threat to pregnant individuals due to pregnancy-induced changes in their immune system. As a result, pregnant women are more susceptible to severe illness and complications from influenza compared to those who are not pregnant [2]. Our study was conducted in the depths of the COVID-19 pandemic-related health system crisis. However, during the COVID-19 pandemic, influenza vaccination was promoted in Greece as a necessary public health measure to manage the COVID-19 pandemic. This included facilitating differential diagnosis and preventing the overloading of hospitals and health services associated with influenza infections. The National Public Health Organization in Greece recommends vaccinating pregnant women with mRNA COVID-19 vaccines at the same time as the general population. Despite the fact that some studies highlight that the COVID-19 pandemic has led to more positive intentions for influenza vaccination globally, we did not record data regarding the COVID-19 vaccine in our study population [17]. 

Our survey study revealed that pregnant participants were well aware of the risks associated with contracting influenza during pregnancy, consistent with previous research showing that the majority of pregnant individuals recognize the heightened danger posed by influenza during pregnancy compared to non-pregnant individuals [18]. This underscores the seriousness of influenza’s impact, as evidenced by the 2009 H1N1 influenza A pandemic, which demonstrated the propensity for severe illness, hospitalization, intensive care unit admissions, and unfortunately, fatalities among pregnant individuals [19]. The third trimester of pregnancy is associated with a higher risk of severe influenza-related outcomes, including preterm labor and a low birth weight, as demonstrated in multiple previous studies [20]. Maternal influenza infection also increases the risk of neonatal complications such as a low birth weight and stillbirth, emphasizing the importance of influenza vaccination during pregnancy. This is especially crucial as there is currently no approved influenza vaccine for infants younger than six months [21].

In a cross-sectional study conducted in Kenya, 68.3% of participants considered the flu vaccine safe during pregnancy, and 60.4% believed that it would protect their baby from infection. In our study, 97% of participants viewed the flu vaccine as safe during pregnancy. In Greece and most European countries, influenza vaccination is recommended and provided free of charge to all pregnant women. However, in some countries like Kenya, only tetanus toxoid is recommended as a maternal vaccine, while influenza vaccines are available in the private sector. Another survey in Kenya found that 83.7% of participants would choose to receive the influenza vaccine if it were offered for free [22]. 

Unlike most previous studies that administered flu shots in the second or third trimester, our study allowed vaccination in any trimester, with a significant number of participants receiving the vaccine during the first trimester. This shift may be due to increased awareness among healthcare professionals that influenza vaccination is safe at any trimester [23,24]. Healthcare providers’ recommendations also play a crucial role in pregnant women’s decision to receive vaccinations, as demonstrated in previous studies [24,25]. 

Our study included participants who received the quadrivalent influenza vaccine (IIV4) during pregnancy. This resulted in similar antibody responses against both influenza A and B strains, differing from previous research where antibody levels against influenza B were lower due to the use of trivalent vaccines [26].

Our findings align with the notion that the transfer of maternal antibodies is most robust in the third trimester of pregnancy [16]. We detected the highest rate of positive results for influenza A among neonates born during this period. Additionally, our study found no statistically significant associations between the demographic and clinical characteristics of pregnant individuals and the presence of antibodies against influenza A and B. Some studies have demonstrated that ethnicity can influence immune responses to vaccination. More specifically, a recent study showed that African Americans aged 30 to 40 have higher levels of H1N1 virus antibodies than Caucasians of the same age [27]. In our study, the majority of participants had Greek ethnicity, and this is reason why we elected not to investigate this association. According to previous studies, the majority of IgG is acquired by the fetus during the last four weeks of pregnancy. It i1111s noteworthy that after the 36th week of gestation, cord blood levels rapidly surge [16,28]. This fact confirms our findings where the highest positive results for influenza A were observed in neonates born in the 37th–42nd week of pregnancy (*p* = 0.021). Positive antibodies were also detected in 70.5% of preterm infants (36–31 weeks of gestation). Furthermore, other studies associated newborn weights of less than 2.5 kg with a lower antibody transfer [29,30]. In our study, birth weight did not show a statistically significant association with the presence of antibodies in our sample.

Additionally, our study revealed that the highest proportion of positive results for influenza A were found in the neonates of women vaccinated during the second trimester (*p* = 0.041). Recent research also supports the impact of vaccination trimester and birth season on antibody titers, with notably higher levels observed in cord blood when mothers were vaccinated during the second or third trimesters [31].

Our study’s strengths include a high response rate, lending credibility to our findings and making them representative. Peripheral blood samples, chosen for their accuracy in measuring antibody values, were used instead of umbilical cord blood. Obtaining maternal consent was challenging, but our results are valuable for neonates born from pregnancies with limited placental capacity for transferring protective antibodies.

Despite the robust response rate, limitations persist, including an uneven distribution of participants across pregnancy trimesters due to the study’s limited sample size. Furthermore, the study could not definitively exclude subclinical influenza cases, and the data collection process relied on non-standardized questionnaires. Additionally, the absence of maternal blood samples prevented the correlation of IgG antibody presence or titers between pregnant women and their neonates. The lack of a quantitative assay to determine antibody titers represents another significant limitation, constraining the depth of our analytical capabilities. Nevertheless, our study supports the hypothesis that maternal immunization through vaccination during pregnancy enhances neonatal immunization against specific pathogens, such as influenza.

Since infants under six months of age cannot receive influenza vaccinations, enhancing humoral immunity through the transplacental transmission of maternal antibodies emerges as a vital preventative measure during pregnancy. Understanding the kinetics of vertically conveyed immunity is crucial, as it provides neonates with a layer of defense against infections like influenza during their early stages of life. Risks associated with influenza infection are neglected; thus, vaccination remains as the primary prevention strategy. Transplacental antibody transport begins early in pregnancy, reaching its peak in the final four weeks of gestation. Consequently, all pregnant individuals, regardless of gestational stage, should be eligible for complimentary influenza vaccination through their respective country’s National Immunization Program. Health policymakers and obstetric care providers should strongly advocate for influenza vaccinations. 

## 5. Conclusions

Pregnancy orchestrates intricate trimester-specific immunological adjustments that are vital for safeguarding the well-being of the maternal–newborn dyad. This inquiry illuminates the determinants impacting neonatal antibody acquisition following maternal influenza vaccination. The profound comprehension of these factors governing placental IgG antibody transference assumes paramount significance, offering avenues for therapeutic manipulation to confer early life advantages to neonates. In summation, amid the escalating global prevalence of infectious maladies, particularly in resource-limited regions, enhancing neonatal survival rates and refining the paradigm of prenatal vaccination is an imperative mandate.

## Figures and Tables

**Table 1:** Demographics, characteristics, and obstetric history of pregnant women enrolled in the study.

Variable	N	%
Age (years)		
<24	1	1.3
24–29	11	14.1
30–34	29	37.2
35–40	31	39.7
>40	6	7.7
Nationality		
Greek	71	91
Other	7	9
Level of education		
High School	17	21.8
Bachelor	46	59
Master	13	16.7
Doctorate	2	2.6
Family status		
Married	67	85.9
Single	11	14.1
Profession (You can choose more than one answer)		
Housewife	6	7.7
Student	1	1.3
Office Clerk	2	2.6
Teacher/Professor	3	3.8
Manager	0	0
Finance employee	0	0
Lawyer	2	2.6
Health professional	3	3.8
Technician	0	0
Business owner	1	1.3
Freelancer	7	9
Private employee	37	47.4
Civil servant	5	6.4
Unemployed	11	14.1
Time period of vaccination against flu in relation to the current pregnancy		
Up to 6 months before the beginning of the current pregnancy	5	6.4
1st trimester	31	39.7
2nd trimester	29	37.2
3rd trimester	13	16.7
Number of previous pregnancies		
0	34	43.6
1	31	39.7
2	12	15.4
>2	1	1.3
Miscarriage history		
Yes	10	12.8
No	68	87.2
History of premature birth		
Yes	14	17.9
No	64	82.1
History of chronic diseases		
Yes	20	25.6
No	58	74.4
Contact with high-risk groups at home		
Yes	31	39.7
No	47	60.3
Contact with high-risk groups in the workplace		
Yes	15	19.2
No	63	80.8
Active smoker		
Yes	3	3.8
No	75	96.2

**Table 2:** Knowledge and attitudes of pregnant women about influenza virus and influenza vaccine.

Variable	N	%
Is the flu a transmissible disease?		
Yes	77	98.7
No	0	0
I don’t know	1	1.3
May complications from the flu require hospitalization?		
Yes	62	79.5
No	3	3.8
I don’t know	13	16.7
Are pregnant women at higher risk for complications from the flu than non-pregnant women?		
Yes	53	67.9
No	1	1.3
I don’t know	24	30.8
Do you know that the flu vaccination is recommended and offered for free for pregnant women in Greece?		
Yes	77	98.7
No	1	1.3
Is the flu vaccine dangerous during pregnancy?		
Yes	0	0
No	77	98.7
I don’t know	1	1.3
Have you been vaccinated against seasonal flu during the period 6 months–5 years before the beginning of the current pregnancy?		
Yes	27	34.6
No	51	65.4
During pregnancy, have you been informed about the flu vaccine and how to obtain it from your doctor, midwife, or other health professional?		
Yes	70	89.7
No	8	10.3

**Table 3:** Association between maternal characteristics and their attitudes towards influenza vaccination.

Variable	Have You Been Vaccinated against Seasonal Influenza during the Period 6 Months–5 Years before the Beginning of the Current Pregnancy?		*p*-Value
Age (years)	No	Yes	0.169
<24	1	0	
24–29	6	5	
30–34	21	8	
35–40	17	14	
>40	6	0	
Level of education	No	Yes	0.960
High School	11	6	
Bachelor	30	16	
Master	9	4	
Doctorate	1	1	
Profession	No	Yes	0.868
Housewife	4	2	
Student	0	1	
Office Clerk	1	1	
Teacher/Professor	2	1	
Manager	0	0	
Finance employee	0	0	
Lawyer	2	0	
Health professional	1	2	
Technician	0	0	
Business owner	1	0	
Freelancer	5	2	
Private employee	25	12	
Civil servant	3	2	
Unemployed	7	4	
History of chronic diseases	No	Yes	0.593
Yes	12	8	
No	39	19	
Contact with high-risk groups at home	No	Yes	0.052
Yes	16	15	
No	35	12	
Contact with high-risk groups in the workplace	No	Yes	0.033
Yes	6	9	
No	45	18	

**Table 4:** Characteristics of the pregnancies and neonates.

Variable	N	%
Neonates’ sex		
Female	43	51.8
Male	40	48.2
Week of birth		
37–42	66	79.5
34–36	15	18.1
<34	2	2.4
Categories of weight at birth		
Small for gestational age <10th percentile	7	8
Appropriate for gestational age 10–90th percentile	73	88
Large for gestational age (LGA) >90th percentile	3	4
Type of delivery		
Caesarean Section	53	67.9
Natural birth	25	32.1
Twin Pregnancy		
Yes	5	6.4
No	73	93.6
Confirmed influenza infection during pregnancy		
Yes	0	0
No	78	100
Neonates’ antibodies against influenza A		
Negative (<9 U)	10	12.1
Positive (>11 U)	66	79.5
Equivocal (9–11 U)	7	8.4
Neonates’ antibodies against influenza B		
Negative (<9 U)	15	18.1
Positive (>11 U)	60	72.3
Equivocal (9–11 U)	8	9.6
The time of year of sampling collection (flu season)		
Yes	46	55.5
No	37	44.6

**Table 5:** Association of pregnant women’s and neonates’ characteristics with the presence of antibodies against influenza A.

Variable	Antibodies against Influenza A			*p*-Value
	Negative	Positive	Equivocal	
Time period of vaccination against flu in relation to the current pregnancy				0.041
Up to 6 months before the beginning of the current pregnancy	2	3	0	
1st trimester	3	30	2	
2nd trimester	2	26	1	
3rd trimester	3	7	4	
Age (years)				0.463
<24	0	1	0	
24–29	1	9	2	
30–34	5	25	0	
35–40	4	25	5	
>40	0	6	0	
Twin pregnancy				0.582
No	8	58	7	
Yes	2	8	0	
Active smoker				0.324
Yes	1	2	0	
No	9	64	7	
Week of birth				0.021
37–42	5	55	6	
34–36	3	11	1	
<34	2	0	0	
Sex				0.341
Females	3	36	4	
Males	7	30	3	
Categories of weight				0.272
Small for gestational age <10th percentile	3	4	0	
Appropriate for gestational age 10–90th percentile	9	59	5	
Large for gestational age (LGA) >90th percentile	0	3	0	
Time between vaccination and blood collection (median value (range), weeks)	23.8 (2–56.6)	21.5 (4–88)	10.3 (1.3–34.2)	0.441

**Table 6:** Association of pregnant women’s and neonates’ characteristics with the presence of antibodies against influenza B.

Variable	Antibodies against Influenza B			*p*-Value
	Negative	Positive	Equivocal	
Time period of vaccination against flu in relation to the current pregnancy				0.060
Up to 6 months before the beginning of the current pregnancy	3	2	0	
1st trimester	7	24	3	
2nd trimester	2	26	2	
3rd trimester	3	8	3	
Age (years)				0.343
<24	1	0	0	
24–29	5	6	2	
30–34	4	24	2	
35–40	5	25	3	
>40	0	5	1	
Twin pregnancy				0.665
No	13	52	8	
Yes	2	8	0	
Active smoker				0.693
Yes	0	3	0	
No	15	57	8	
Week of birth				0.281
37–42	10	48	8	
34–36	4	11	0	
<34	1	1	0	
Sex				0.594
Females	7	33	3	
Males	8	27	5	
Categories of weight				0.242
Small for gestational age < 10th percentile	1	5	1	
Appropriate for gestational age 10–90th percentile	11	54	8	
Large for gestational age (LGA) > 90th percentile	2	1	0	
Time between vaccination and blood collection (median value (range), weeks)	30 (1.3–88)	18.4 (3–56.6)	18.85 (3–32.2)	0.047

**Table 7:** Concordant IgG detection results among twin neonates.

Twin Pregnancy		Influenza A	Influenza B
1	Twin 1	+	+
	Twin 2	+	+
2	Twin 1	−	+
	Twin 2	−	+
3	Twin 1	+	−
	Twin 2	+	−
4	Twin 1	+	+
	Twin 2	+	+
5	Twin 1	+	+
	Twin 2	+	+

## Data Availability

The datasets produced and analyzed in the present study are not openly accessible to the public due to privacy and confidentiality concerns. Nevertheless, they can be obtained from the corresponding author upon reasonable request.
